# Chromogranin-A production and fragmentation in patients with Takayasu arteritis

**DOI:** 10.1186/s13075-016-1082-2

**Published:** 2016-08-17

**Authors:** Enrico Tombetti, Barbara Colombo, Maria Chiara Di Chio, Silvia Sartorelli, Maurizio Papa, Annalaura Salerno, Enrica Paola Bozzolo, Elisabetta Tombolini, Giulia Benedetti, Claudia Godi, Chiara Lanzani, Patrizia Rovere-Querini, Alessandro Del Maschio, Alessandro Ambrosi, Francesco De Cobelli, Maria Grazia Sabbadini, Elena Baldissera, Angelo Corti, Angelo A. Manfredi

**Affiliations:** 1 Department of Medicine and Division of Immunology, Transplantation & Infectious Diseases, IRCCS San Raffaele Scientific Institute, via Olgettina 60, 20132 Milan, Italy; 2 Vita-Salute San Raffaele University, 20132 Milan, Italy; 3 Division of Oncology, IRCCS San Raffaele Scientific Institute, 20132 Milan, Italy; 4 Department of Radiology, IRCCS San Raffaele Scientific Institute, 20132 Milan, Italy; 5 Department of Neuroradiology, IRCCS San Raffaele Scientific Institute, 20132 Milan, Italy; 6 Genomics of Renal Disease and Hypertension Unit, IRCCS San Raffaele Scientific Institute, 20132 Milan, Italy

**Keywords:** Takayasu arteritis, Biomarker, Chromogranin A, Vasculitis, Vascular remodelling, Proton-pump inhibitors

## Abstract

**Background:**

Chromogranin-A (CgA) is a secretory protein processed into peptides that regulate angiogenesis and vascular cells activation, migration and proliferation. These processes may influence arterial inflammation and remodelling in Takayasu arteritis (TA).

**Methods:**

Plasma levels of full-length CgA (CgA_439_), CgA fragments lacking the C-terminal region (CgA-FRs) and the N-terminal fragment, CgA_1–76_ (vasostatin-1, VS-1) were analysed in 42 patients with TA and 20 healthy age-matched controls. Vascular remodelling was longitudinally assessed by imaging. CgA peptides were related to markers of systemic and local inflammation, disease activity and vascular remodelling.

**Results:**

Levels of CgA-FRs and VS-1 were increased in TA. Treatment with proton-pump inhibitors (PPIs) and arterial hypertension partially accounted for CgA levels and high inter-patient variability. CgA_439_, CgA-FRs and VS-1 levels did not reflect disease activity or extent. Markers of systemic or local inflammation correlated with higher CgA-FRs and VS-1 in normotensive patients and with higher CgA_439_ in hypertensive patients. Treatment with non-biologic anti-rheumatic agents was associated with increased CgA-FRs and a distinctive regulation of CgA processing. Reduced blood levels of anti-angiogenic CgA peptides were associated with vascular remodelling in the groups of patients on PPIs and with arterial hypertension.

**Conclusions:**

The plasma levels of CgA fragments are markedly increased in TA as a consequence of disease- and therapy-related variables. Anti-angiogenic forms of CgA may limit vascular remodelling. Given the effect of the various CgA peptides, it is advisable to limit the therapeutic prescriptions that might influence CgA-derived peptide levels to clearly agreed medical indications until further data become available.

**Electronic supplementary material:**

The online version of this article (doi:10.1186/s13075-016-1082-2) contains supplementary material, which is available to authorized users.

## Background

Takayasu arteritis (TA) is a rare idiopathic chronic-relapsing large vessel vasculitis, usually affecting young women and associated with considerable morbidity and mortality [1–4]. Typical arterial lesions affect the aorta and its major branches, with wall thickening and possible remodelling resulting either in steno-occlusion or in dilatation up to aneurysm formation [1, 4, 5]. Limiting the progression of vascular lesions is an important therapeutic target in TA [6] as vascular complications have a prognostic impact [7]. However, we lack reliable biomarkers to assess disease activity and vascular progression. Despite TA being an inflammatory disease, the relationship between systemic inflammation and vascular progression is loose, as about 60 % of patients believed to be in remission and without evidence of systemic inflammation develop new arterial lesions on serial angiographic studies [5]. Local inflammation and/or pauci-inflammatory vascular remodelling might be even relevant in the evolution of lesions [8–11] in the context of a long-lasting injury caused by immune response against vascular antigens [11]. Indeed, intimal hyperplasia, which is a stereotyped remodelling response to many vascular injuries, is a typical finding in TA lesions and contributes to wall thickening. Locally generated or released growth factors such as fibroblast growth factor and platelet-derived growth factor might contribute to intimal hyperplasia. Vasa vasorum neoangiogenesis and migration/proliferation of medial vascular smooth muscle cells (VSMCs) are associated with intimal hyperplasia and arterial remodelling in giant cell arteritis (GCA), a large vessel vasculitis cognate of TA [11, 12].

Chromogranin-A (CgA) is a protein stored in secretory granules of many neuroendocrine cells, neurons, granulocytes and cardiomyocytes [13, 14]. CgA has a crucial intracellular role in secretory granule biogenesis and calcium homeostasis [14]. Tissue-specific and context-specific proteolytic cleavage of CgA yields polypeptides with paracrine and endocrine activity (hence referred as the CgA system) [14, 15]. At least four CgA polypeptides containing the N-term region have been detected in the blood of healthy subjects, including full-length CgA protein (or CgA_439_), large fragments cleaved after residue 436, various polypeptides spanning from the N-terminus to the central region but lacking the C-terminal region (CgA-FRs) and the N-terminal peptide CgA_1–76_, alias vasostatin-1 (VS-1) [15]. CgA_439_, CgA-FRs and VS-1 differentially modulate angiogenesis [15]. It has been recently shown that CgA contains a functional anti-angiogenic site in the C-terminal region 410–439 [15]. Thus fragments lacking the C-terminal regions (CgA-FRs) are devoid of anti-angiogenic activity. In addition, CgA contains a latent anti-angiogenic site in the N-terminal region 1–76 and a pro-angiogenic site in the region 352–372, which can be activated by the proteolytic cleavage of Q76–K77 and R373–R374, respectively [15]. Accordingly, CgA_439_ and VS-1 inhibit angiogenesis in various angiogenic assays, whereas CgA_1–373_ (one of the CgA-FRs) and the CgA-FRs produced by thrombin digestion of CgA can induce the secretion of fibroblast growth factor-2 (FGF2) from endothelial cells and stimulate angiogenesis [15, 16]. The CgA-dependent regulation of the angiogenic switch has been demonstrated in the bone marrow of patients with multiple myeloma and experimental murine models of the disease further strengthening the contention that the balance between pro- and anti-angiogenic CgA-derived peptides has important physiopathological consequences [17].

CgA-related polypeptides can also influences fibroblast adhesion, endothelial and VSMC proliferation and migration, endothelial response to inflammatory stimuli, cardiac function and vascular tone [14–16, 18–23]. Furthermore, CgA_439_ accelerates wound healing in mice in a process dependent on the integrin α_v_β_6_ [24]. Based on these evidences, it has been hypothesized that local and systemic changes in the concentrations of CgA and its fragments may contribute to the homeostatic vascular regulation in normal conditions as well as of vascular inflammation and remodelling in response to injury.

Increased blood levels of CgA have been shown in numerous inflammatory and non-inflammatory conditions, including neuroendocrine tumours, renal failure, arterial hypertension, chronic heart failure and rheumatoid arthritis [18, 25]. Concurrent therapy can also influence CgA levels: for example, proton-pump inhibitors (PPIs, frequently used in patients on steroid therapy) increase the circulating levels of CgA by inducing hyperplasia of the gastric entherochromaffin cells. CgA is particularly elevated in patients with refractory GCA and might represent a marker of smouldering disease [26].

Here, we assessed the plasmatic levels of CgA_439_, CgA-FRs and VS-1 and the relative proportions (ratios of each peptide to total CgA, CgA_tot_) in TA. To understand the link with arterial remodelling, we verified whether their concentrations correlate with inflammatory and non-inflammatory features of TA.

## Methods

### Study sample

Fifty-one patients fulfilling the American College of Rheumatology (ACR) criteria for TA [27] were evaluated in 2013 at the San Raffaele Scientific Institute in Milan. All patients were extensively evaluated to exclude TA mimics, as previously described [10]. We performed a cross-sectional analysis of disease biomarkers between April and August 2013, excluding nine patients that did not attend a visit in that period or had moderate to severe renal or heart failure, and carried out a longitudinal evaluation of arterial involvement as assessed by serial radiological imaging follow-up (see below). The final sample consisted of 42 consecutive patients with a median age of 46 years (range 23 to 66 years). Twenty age-matched women served as healthy controls (HCs). All subjects gave written informed consent for participation in the study and the Institutional Review Board of the San Raffaele University Hospital (Comitato Etico dell’Ospedale San Raffaele, Milano, Italy) approved the study protocol (protocol “Autoimmuno-mol”, PI Angelo Manfredi). This study was conducted in accordance with the Declaration of Helsinki.

### Clinical assessment and laboratory biomarkers

Systemic activity of TA was evaluated according to National Institutes of Health (NIH) criteria [5]. Arterial hypertension was defined in accordance with international guidelines. Cardiac involvement was defined as significant aortic regurgitation or presence of systolic or diastolic dysfunction. Three inflammatory reactants were evaluated: erythrocyte sedimentation rate (ESR), and concentration of C-reactive protein (CRP) and of pentraxin-3 (PTX3). ESR was assessed by the Westergren method, CRP by a latex-enhanced immuno-turbidimetric assay (ADVIA Chemistry System, Bayer AG, Leverkusen Germany), and plasma PTX3 by ELISAs as previously described [10]. Four sandwich ELISAs were used to detect CgA-derived polypeptides (Additional file 1: Figure S1) [15]: (1) 439 ELISA, that specifically detects CgA_439_; (2) 436/439 ELISA, that detects the peptides with the N-terminal domain and the C-terminal region, with or without the 436–439 residues; (3) 436/439 + FRs-ELISA, that detects peptides with the N-terminal domain and the central region; (4) 76 ELISA, that detects VS-1. CgA_tot_ was computed summing the results of 436/439 + FRs-ELISA and 76 ELISA, thus including all the CgA-derived polypeptides containing the N-terminal region. Given that both CgA_439_ and VS-1 have anti-angiogenic properties, we pooled these two polypeptides by summing the ranks of the respective peptides in our sample. The levels of CgA-FRs were computed subtracting the results of 436/439 + FRs ELISA and 436/439 ELISA. To study the regulation of CgA processing, we evaluated the ratios of CgA_439_/CgA_tot_, CgA-FRs/CgA_tot_ and VS-1/CgA_tot_. There were no samples missing for CRP, PTX3 and CgA or derived peptides assessment. Three samples were missing for ESR. Samples were analysed in a random order and in a blinded manner.

### Imaging assessment

All patients were regularly monitored with magnetic resonance angiography (MRA) and vascular ultrasonography (US). Two patients had contraindications to MRA and underwent computed tomography angiography (CTA). MRA was performed with a 1.5-T magnetic resonance whole body scanner (Achieva Nova Master; Philips Medical Systems, Best, The Netherlands) with phased-array head and neck or phased-array thoracic dedicated coils. Morphologic sequences [proton-density weighted (PD) black blood turbo spin echo (TSE): field of view (FOV) 260 × 152; acquisition (Acq) matrix = 260 × 264; reconstruction (Recon) matrix = 528; Acq voxel MPS = 1.00/0.95/6.00; Recon voxel MPS = 0.49/0.49/600; echo time/repetition time (TE/TR) = 20/2 beats; electrocardiogram (ECG) triggered; in expiratory breath hold of approximately 10 sec] were performed to evaluate vessel wall thickening. First-pass MRA dynamic sequences targeted on thoraco-abdominal aorta and supra-aortic trunks (FOV 450 × 390; Acq matrix = 352 × 200; Recon matrix = 670; Acq voxel = 1.28/1.90/3.00; Rec voxel = 0.67/0.67/1.5; slice thickness = 1.5 mm; TR/TE/α = 4.6/1.35/40; acquisition time = 21 sec) were performed during contrast media infusion (Gadovist®: 1 nmol/ml Bayer Pharma, Berlin, Germany). High-resolution (HR) sequences were performed before and after contrast administration [coronal HR three-dimensional (3D) fast field echo (FFE): FOV 360 × 276; Acq matrix = 684 × 521; Rec matrix 880; slice thickness = 0.8 mm; TR/TE/flip angle (FA) = 6.3/2.1/20; acquisition time = 3:28 min]. These sequences allow the evaluation of vessel wall thickness up to 1 mm. CTA was performed with a Brilliance CT 64-channel scanner (Philips Medical Systems, Best, The Netherlands) with administration of 130 ml of Ultravist 370 (Bayer Healthcare LCC, Leverkusen, Germany) at a flow rate of 5 ml/sec. US was performed with iU22 Matrix Ultrasound system (Philips Medical Systems). US and CTA evaluations assessed carotid, subclavian, abdominal and femoral districts, while MRA was focused on thoraco-abdominal or cervico-thoracic regions according to disease involvement. Lesions were evaluated by two radiologists with an expertise in cardio-vascular imaging, blinded to the clinical status of patients. Variables evaluated for each lesion at MRA and CTA included lesion width, length, residual lumen and contrast enhancement. Since gadofosveset trisodium was retired, the presence or absence of vessel wall enhancement was scored by two radiologist experts in cardio-vascular imaging, blinded to the clinical status of patients. Enhancement data were available for 30 patients. At US, lesions width, residual lumen, peak systolic blood flow velocity and the presence of the halo sign were assessed. Imaging follow-up occurred yearly or, in case of relapses, until disease enters the tardive phase, clinical and morphological stability for at least 3–5 years.

Progression in arterial involvement was defined as appearance of novel lesions or as increase in width and/or length and/or percentage of luminal stenosis of established vasculitic lesions at follow-up. Two patients were evaluated with imaging only at a single time point, and progression over time could not be assessed. Progressive enlargement of ectasias/aneurysms may be related to biomechanical factors independent of disease activity. Therefore, it has not been included in the definition of progression of arterial involvement. Similarly, progression due to concurrent atherosclerosis was not taken into account. Confounder atherosclerotic lesions were identified and excluded on the basis of typical sites (arterial branches), eccentric appearance of the lesions, short and focal lesions, inhomogeneous content and irregular luminal border on US, the presence of parietal calcifications on the luminal side of the lesion and the absence of halo signs or adventitial thickenings on US.

### Statistical analysis

We expressed scalar variables as median values and ranges. Mann-Whitney *U* test was used to compare biomarkers between patients with TA and controls, or between various subgroups of patients with TA stratified according to the presence or the absence of therapy with PPIs, steroids and immunosuppressive agents, arterial hypertension, wall enhancement, vascular progression and active disease. Multivariate analysis with multi-factor analysis of variance (ANOVA) was performed to verify the relationship between stratifying variables and plasma levels of CgA fragments. Plasma levels of CgA_439_, CgA-FRs and VS-1, their *ratio* to CgA_tot_ and the anti-angiogenic CgA potential were used as dependant variables of the analysis. Five candidate factors were considered in the model on the basis of their clinical relevance and of their potential involvement: therapy with PPIs, presence of arterial hypertension, vascular progression, therapy with prednisone and therapy with immunosuppressive agents. Considering the sample size, we set the optimal number of factors in the model at four to avoid over-parameterization and loss of statistical power. Given the evidence of the impact of therapy with PPIs on plasma CgA levels [28] and the association between treatment with PPI and with steroids in our sample (*p* < 0.001), the latter was excluded from the final model, resulting in a four-way ANOVA. Kruskal-Wallis test was used to compare values of biomarkers within various classes of arterial involvement [29]. Spearman rank correlation coefficient was calculated for the correlation analyses. Associations were evaluated with χ^2^ test and Fisher exact test.

A two-tailed *p* value less than 0.05 was considered to represent statistically significant differences, and *p* values less than 0.10 were shown in the tables. Statistical analysis was performed with IBM SPSS Statistics, version 20 (IBM Corp., Armonk, NY, USA).

## Results

### Patient characteristics

Table 1 summarizes the demographic, clinical and laboratory characteristics of patients with TA (42 subjects, 39 women and three men) and of age-matched HCs (20 women). The median age at TA onset was 30 years (range 17–56 years). Thirty-seven (88 %) TA patients had a widespread diffuse arterial involvement (angiographic class II or V). Sixteen patients (38 %) had arterial aneurysms. Thirty-eight patients (90 %) were on treatment: 30 received steroids, 30 immunosuppressive agents (12 azathioprine, 11 methotrexate, four mofetil mycophenolate, two sirolimus, one cyclophosphamide), 16 tumour necrosis factor (TNF) blockers, two tocilizumab and one rituximab. Thirty patients were on treatment with proton-pump inhibitors (PPIs). Twelve patients (29 %) fulfilled the NIH criteria for active TA. Arterial wall enhancement was detectable in 16 % (5/30) and vascular progression in 22 % (9/40) of the patients. Twenty-two (52 %) patients had arterial hypertension. CRP and PTX3 concentrations were higher in patients with TA (2.6 mg/l, 0.1–40 mg/l and 5.5 ng/ml, 1.3–55 ng/ml, respectively) than in HCs (0.6 mg/l, 0.3–9.0 mg/l, *p* = 0.017 and 3.9 ng/ml, 1.4–6.5 ng/ml, *p* = 0.009 respectively).

**Table 1:** Characteristics of the TA patients and HCs

	HCs	TA (N = 42)	*p* value
Qualitative variables			
Sex (F:M)	20:0	39:3	n.s.
Class of arterial involvement:	N.E.		
1 2A 2B 3 4 5	- - - - - -	4 (10 %) 4 (10 %) 3 (7 %) 1 (2 %) 0 30 (71 %)	- - - - - -
Coronary involvement	N.E.	6 (14 %)	-
Pulmonary artery involvement	N.E.	13 (31 %)	-
Aneurysms	0	16 (38 %)	-
Steroids	0	30 (71 %)	-
Immunosuppressive therapy: Azathioprine Methotrexate Mycophenolate Sirolimus Cyclophosphamide	0 - - - - -	30 (71 %) 12 (29 %) 11 (26 %) 4 (10 %) 2 (5 %) 1 (2 %)	- - - - - -
Biologic therapy: TNF blockers Tocilizumab Rituximab	0 - - -	19 (45 %) 16 (38 %) 2 (5 %) 1 (2 %)	- - - -
Active disease (NIH criteria)	N.E.	12 (29 %)	-
Anticoagulants	0	7 (17 %)	-
Arterial hypertension	0	22 (52 %)	-
Cardiac involvement	N.E.	12 (29 %)	-
Vascular enhancement (N = 30)	N.E.	5 (16 %)	-
Vascular progression (N = 40)	N.E.	9 (22 %)	-
Scalar variables (median, range)			
Age at disease onset (years)	N.E.	30 (17–56)	-
Disease duration (years)	N.E.	10 (0-34)	-
Creatinine (mg/dl)	N.A.	0.70 (0.44–1.60)	-
PDN dose (mg/day; N = 30)	0	5 (3–35)	-
N vessels	N.E.	4 (1–7)	-
ESR (mm/h)	N.A.	15 (1–78)	-
Serum CRP (mg/l)	0.6 (0.3–9.0)	2.6 (0.03–40)	0.017
Plasma PTX3 (ng/ml)	3.9 (1.4–6.5)	5.5 (1.3–55)	0.009
CgA_tot_ (nM)	0.98 (0.47–1.72)	2.36 (0.45–7.85)	0.001
CgA_439_ (nM)	0.04 (0–0.07)	0.05 (0-0–.78)	n.s.
CgA-FRs (nM)	0.64 (0.22–1.17)	1.63 (0.22–6.68)	0.001
VS-1 (nM)	0.10 (0.07–0.50)	0.20 (0.02–1.15)	0.020
CgA_439_/CgA_tot_	5 % (0–17 %)	1 % (0–24 %)	n.s.
CgA-FRs/CgA_tot_	67 % (46–88 %)	66 % (47–94 %)	n.s.
VS-1/CgA_tot_	13 % (7–29 %)	11 % (1–27 %)	n.s.

Quantitative and qualitative variables related to TA were evaluated in TA patients and healthy controls (HCs). N vessels refers to the number of vessels involved by the disease (see “Methods”)

*TA* Takayasu arteritis, *n.s.* not significant, *TNF* tumour necrosis factor, *N.E*. not evaluable, *N.A.* not available, *PDN* prednisone, *ESR* erythrocyte sedimentation rate, *CRP* C-reactive protein, *PTX3* pentraxin-3, *CgA*_*tot*_ total chromogranin-A, *CgA*_*439*_ full-length CgA (residues 1–439), *CgA-FRs* fragments of CgA spanning from the N-terminus to the central region but lacking the C-terminal region, *VS-1* vasostatin-1

### CgA levels in TA

**Fig. 1:**
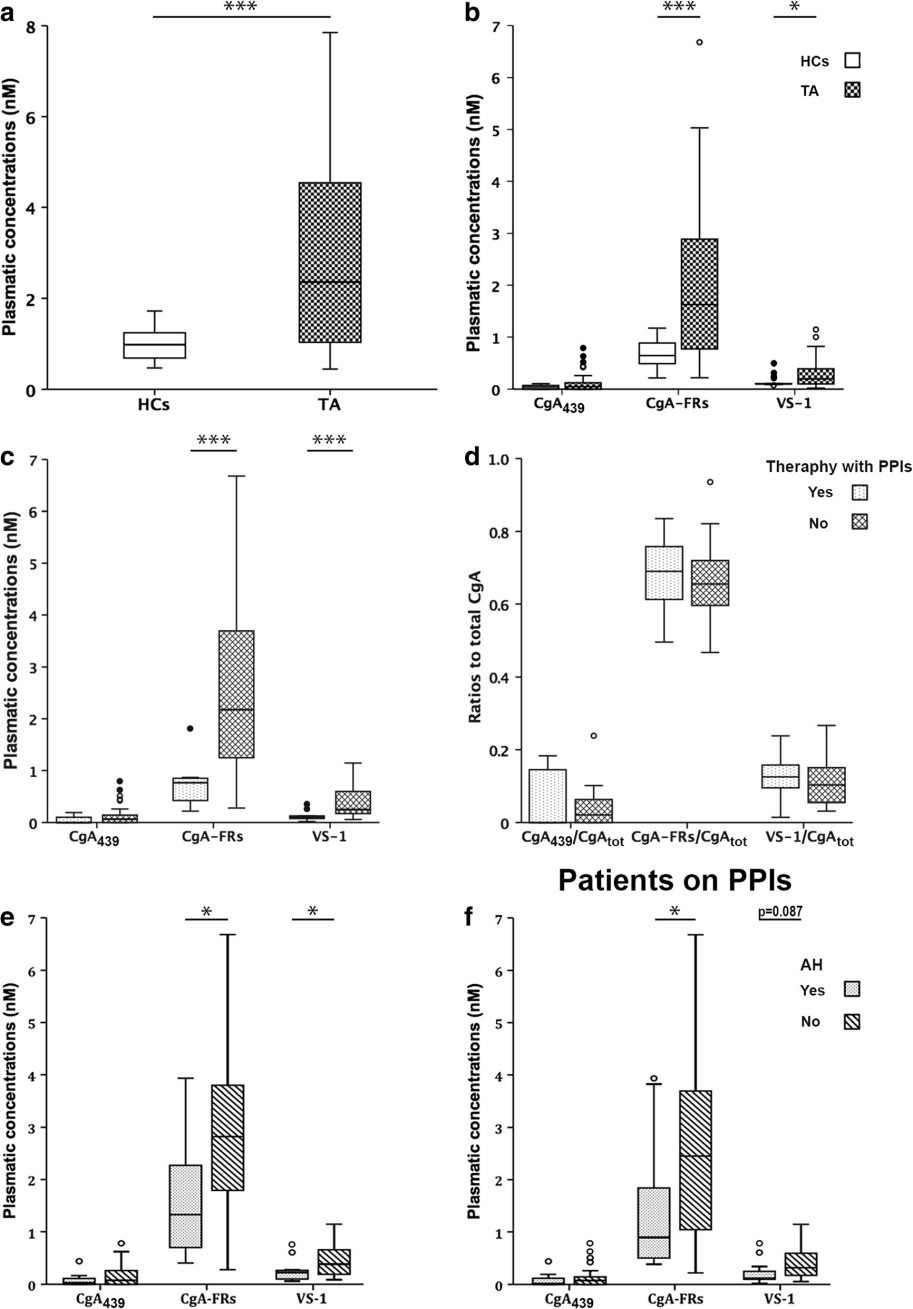
Levels of CgA-derived polypeptides in patients with TA. **a** Plasma concentrations of total CgA, obtained summing the concentration of the full-length molecule with that of the various CgA fragments in patients with TA and HCs (see “Methods”). ^***^: significantly different from HCs, *p* ≤ 0.001. **b** Plasma concentrations of CgA_439_, CgA-FRs and VS-1 in patients with TA and HCs. ^***^ and ^*^: significantly different from HCs, *p* ≤ 0.001 and *p* ≤ 0.05 respectively. **c** Plasma concentrations of CgA_439_, CgA-FRs and VS-1 in TA patients with or without PPIs. ^***^: significantly different from patients without PPIs, *p* ≤ 0.001. **d** Ratios of CgA_439_, CgA-FRs and VS-1 to CgA_tot_ in TA patients with or without PPIs. **e**-**f** Plasma concentrations of CgA_439_, CgA-FRs and VS-1 in normotensive versus hypertensive patients either in the whole group of TA patients (*panel* **e**) and in those on PPIs (*panel* **f**). ^*^: significantly different from normotensive patients. *AH* arterial hypertension, *CgA*_*439*_ full-length chromogranin-A (residues 1–439), *CgA-FRs*, fragments of CgA spanning from the N-terminus to the central region but lacking the C-terminal region, *CgA*_*tot*_ total CgA, *HC* healthy controls, *PPI* proton-pump inhibitors, *TA* Takayasu arteritis, *VS-1* vasostatin-1

The CgA system encompasses a family of variably processed polypeptides. We estimated total CgA (CgA_tot_) concentration by assessing all the polypeptides containing the N-terminal region, i.e. by summing the results of 436/439 + FRs ELISA and 76 ELISA [15]. CgA_tot_ was higher in patients with TA than in HCs (2.36 nM, range 0.45 to 7.85 nM vs 0.98 nM, range 0.47 to 1.72 nM, *p* = 0.001; Fig. 1a and Table 1). Similarly, TA patients had higher plasmatic CgA-FRs and VS-1 than HCs (*p* = 0.001 and 0.020, respectively; Fig. 1b and Table 1). The inter-subject variability of the levels of CgA-derived polypeptides was high, especially in the TA group and for CgA_439_ levels (Fig. 1a-b and Table 1), which had the highest coefficient of variation (not shown).

We evaluated disease-related and therapy-related variables. TA patients on PPIs (30/40, 75 %) had significantly more active disease (*p* = 0.009) and were more frequently treated with steroids than patients without PPIs (*p* < 0.001, Table 2). Levels of CgA_tot_ were higher in the group on PPIs. Specifically, patients on PPIs had higher concentrations of CgA-FRs and VS-1 (Fig. 1c and Table 2). High inter-patient variability in the levels of CgA_tot_, CgA_439_, CgA-FRs and VS-1 persisted after stratification for PPI treatment (Fig. 1c and Table 2). CgA_439_/CgA_tot_, CgA-FRs/CgA_tot_ and VS-1/CgA_tot_ ratios were similar in patients whether or not on PPI therapy (Fig. 1d and Table 2). These data suggest that PPIs apparently do not influence CgA processing and only partially account for the high inter-patient variability observed.

**Table 2:** Characteristics of the TA patients stratified for therapy with PPIs and for the presence of arterial hypertension

	Therapy with PPIs			Arterial hypertension		
	No (N = 12)	Yes (N = 30)	*p* value	No (N = 20)	Yes (N = 22)	*p* value
Qualitative variables						
Sex (F:M)	11:1	28:2	n.s.	20:0	19:3	n.s.
Class of arterial involvement:						
1 2A 2B 3 4 5	1 2 1 0 0 8	3 2 2 1 0 22	n.s. n.s. n.s. n.s. N.A. n.s.	2 3 1 1 0 13	2 1 2 0 0 17	n.s. n.s. n.s. n.s. N.A. n.s.
Aneurysms	6	10	n.s.	7	9	n.s.
Steroids	3	27	<0.001	13	17	n.s.
Immunosuppressive therapy:	7	23	n.s.	11	19	0.04
Biologic therapy: TNF blockers Tocilizumab	4 3 1	15 13 1	n.s. n.s. n.s.	10 9 1	9 7 1	n.s. n.s. n.s.
Active disease (NIH criteria)	0	12	0.009	5	6	n.s.
Anticoagulants	3	4	n.s.	3	4	n.s.
Arterial hypertension	4	18	n.s.	N.E.	N.E.	
Therapy with PPIs	N.E.	N.E.				
Cardiac involvement	4	8	n.s.	6	6	n.s.
Vascular enhancement (N = 30)	1	4	n.s.	3	2	n.s.
Vascular progression (N = 40)	1	8	n.s.	5	4	n.s.
Scalar variables (median and range)						
Age (years)	51 (31–66)	41 (23–62)	0.060	40 (23–66)	47 (26–65)	n.s.
Age at TA onset (years)	36 (21–56)	28 (17–56)	n.s.	29 (17–56)	30 (18–56)	n.s.
Disease duration (years)	12 (4–21)	9 (0–34)	n.s.	9 (2–21)	11 (0–34)	n.s.
Creatinine (mg/dl)	0.70 (0.44–1.06)	0.70 (0.56–1.60)	n.s.	0.67 (0.49–0.89)	0.75 (0.44–1.61)	0.084
PDN dose (mg/day)	0 (0–12.5)	5 (0–35)	<0.001	5 (0–25)	5 (0–35)	n.s.
N vessels	4 (1–6)	4 (1–7)	n.s.	3 (1–-6)	4 (1–7)	n.s.
ESR (mm/h)	9 (1–23)	18 (2–78)	0.059	13 (2–73)	18 (1–78)	n.s.
Serum CRP (mg/l)	1.5 (0.1–12)	3.0 (0.3–40)	0.104	2.0 (0.1-40)	2.6 (0.3–36.8)	n.s.
Plasma PTX3 (ng/ml)	4.1 (1.3–44)	5.9 (2.2–55)	n.s.	5.8 (2.5–43.6)	5.3 (1.3–55.0)	n.s.
CgA_tot_ (nM)	1.02 (0.45–2.66)	3.40 (0.60–7.85)	<0.001	1.35 (0.5–3-6.31)	3.76 (0.45–7.85)	0.021
CgA_439_ (nM)	0 (0-0–19)	0.07 (0–0.78)	n.s.	0.01 (0–0.44)	0.07 (0–0.78)	n.s.
CgA-FRs (nM)	0.76 (0.22–1.82)	2.18 (0.28–6.68)	<0.001	0.89 (0.39–3.93)	2.45 (0.22–6.68)	0.030
VS-1 (nM)	0.10 (0.02–0.34)	0.25 (0.06–1.15)	0.001	0.11 (0.02–0.79)	0.32 (0.05–1.15)	0.024
CgA_439_/CgA_tot_	0 % (0–18 %)	2 % (0–24 %)	n.s.	0 % (0–18 %)	2 % (0–24 %)	n.s.
CgA-FRs/CgA_tot_	69 % (50–84 %)	66 % (47–94 %)	n.s.	69 % (50–84 %)	66 % (47–94 %)	n.s.
VS-1/CgA_tot_	12 % (1–24 %)	10 % (3–27 %)	n.s.	12 % (1–24 %)	10 % (3–27 %)	n.s.
Rank CgA_439_+ rank VS-1	27 (5–50)	41 (18–83)	0.002	32 (5–64)	44 (8–83)	0.023

Quantitative and qualitative variables related to TA were evaluated, verifying the impact of the treatment with PPIs and of arterial hypertension. N vessels refers to the number of vessels involved by the disease (see “Methods”)

*TA* Takayasu arteritis, *PPI* proton-pump inhibitor, *n.s.* not significant, *N.A.* not available, *TNF* tumour necrosis factor, *N.E*. not evaluable, *PDN* prednisone, *ESR* erythrocyte sedimentation rate, *CRP* C-reactive protein, *PTX3* pentraxin-3, *CgA*_*tot*_ total chromogranin-A, *CgA*_*439*_ full-length CgA (residues 1–439), *CgA-FRs* fragments of CgA spanning from the N-terminus to the central region but lacking the C-terminal region, *VS-1* vasostatin-1

### Arterial hypertension is associated with higher CgA-FRs and VS-1 in TA

Twenty-two patients had arterial hypertension. Hypertensive patients were more often treated with non-biologic immunosuppressive agents and showed a trend towards higher creatinine levels (Table 2). Levels of CgA-FRs and VS-1 were higher in hypertensive TA patients (*p* = 0.030 and 0.024, respectively, Fig. 1e and Table 2). To take into account the possible confounding effect of PPIs, we repeated the analysis in TA patients on PPIs, observing significantly higher concentrations of CgA-FRs (*p* = 0.035) and a trend toward increased VS-1 (*p* = 0.087, Fig. 1f and Additional file 2: Table S1). Similar CgA_439_/CgA_tot_, CgA-FRs/CgA_tot_ and VS-1/CgA_tot_ were observed in the presence or the absence of arterial hypertension in the whole sample (Table 2) and after stratification for PPI therapy (Additional file 2: Table S1).

### CgA_439_, CgA-FRs and VS-1 differentially reflect systemic and local inflammation in normotensive and hypertensive TA patients

**Fig. 2:**
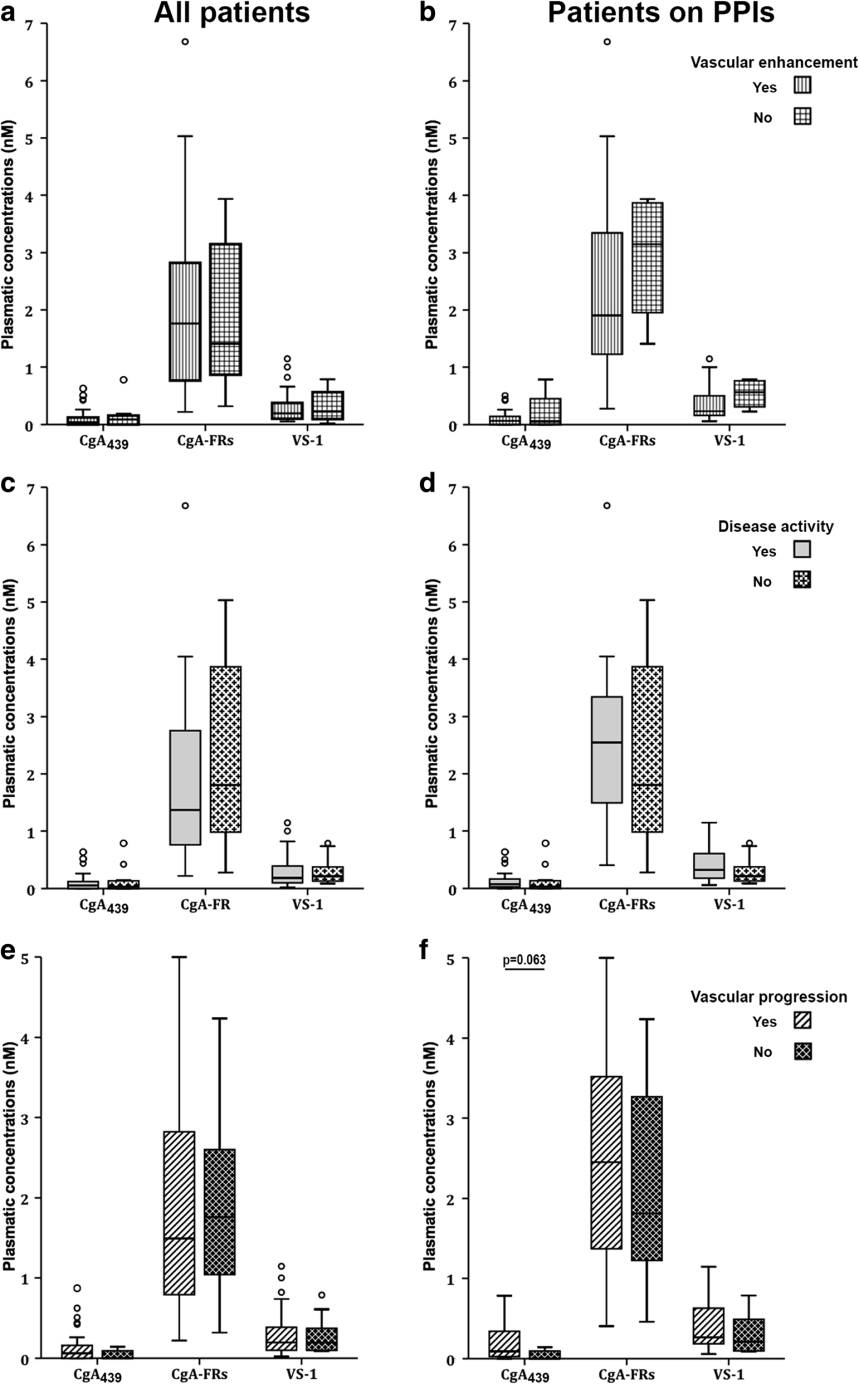
Levels of CgA peptides and selected TA clinical features. Plasma concentrations of CgA_439_, CgA-FRs and VS-1 in the whole group of TA patients (*left panels*) and in those on PPI therapy (*right panels*), stratified for vascular enhancement (*upper panels*), disease activity (*middle panels*) and vascular progression (*lower panels*) (see “Methods”). *CgA*_*439*_ full-length chromogranin-A (residues 1–439), *CgA-FRs* fragments of CgA spanning from the N-terminus to the central region but lacking the C-terminal region, *PPI* proton-pump inhibitors, *VS-1* vasostatin-1

Plasma concentration of CgA-derived polypeptides did not reflect systemic inflammation (as measured by ESR and CRP) or local inflammation (as assessed by PTX3 levels [9] and/or by the presence of arterial wall enhancement at imaging). In particular, ESR and PTX3 did not correlate with the levels of CgA_439_, CgA-FRs, VS-1, CgA_439_/CgA_tot_, CgA-FRs/CgA_tot_ and VS-1/CgA_tot_ in TA patients, even after stratification for PPI therapy (Table 3). CRP concentrations correlated with CgA-FRs but the correlation was lost after stratification for PPI treatment. Levels of CgA_439_, CgA-FRs and VS-1 (Fig. 2a-b), and their ratios to CgA_tot_ were similar in patients with or without arterial wall enhancement at imaging.

**Table 3:** Correlations of the CgA peptides in TA patients with markers of systemic and local inflammation and with the number of involved vessels

Whole TA group (N = 42)				
	CgA_439_	CgA-FRs	VS-1	CgA_tot_
ESR	0.179 n.s.	0.181 n.s.	0.173 n.s.	0.158 n.s.
CRP	0.015 n.s.	0.326 *p* = 0.035	0.229 n.s.	0.300 *p* = 0.053
PTX3	-0.232 n.s.	-0.082 n.s.	0.291 *p* = 0.062	0.085 n.s.
N vessels	0.196 n.s.	0.039 n.s.	-0.047 n.s.	0.066 n.s.
	CgA_439_/CgA_tot_	CgA-FRs/CgA_tot_	VS-1/CgA_tot_	
ESR	0.134 n.s.	0.178 n.s.	0.016 n.s.	
CRP	-0.009 n.s.	0.088 n.s.	-0.123 n.s.	
PTX3	-0.022 n.s.	-0.123 n.s.	0.154 n.s.	
N vessels	0.153 n.s.	-0.176 n.s.	-0.178 n.s.	
TA on PPIs (N = 30)				
	CgA_439_	CgA-FRs	VS-1	CgA_tot_
ESR	0.119 n.s.	0.045 n.s.	0.010 n.s.	0.002 n.s.
CRP	0.030 n.s.	0.140 n.s.	0.028 n.s.	0.142 n.s.
PTX3	-0.258 n.s.	0.065 n.s.	0.176 n.s.	0.071 n.s.
N vessels	0.309 *p* = 0.096	0.017 n.s.	-0.009 n.s.	0.076 n.s.
	CgA_439_/CgA_tot_	CgA-FRs/CgA_tot_	VS-1/CgA_tot_	
ESR	0.174 n.s.	0.319 *p* = 0.097	-0.009 n.s.	
CRP	0.106 n.s.	0.200 n.s.	-0.169 n.s.	
PTX3	-0.241 n.s.	0.037 n.s.	0.133 n.s.	
N vessels	0.251 n.s.	-0.194 n.s.	-0.138 n.s.	
Normotensive TA (N = 20)				
	CgA_439_	CgA-FRs	VS-1	CgA_tot_
ESR	-0.368 n.s.	-0.028 n.s.	0.415 *p* = 0.110	-0.162 n.s.
CRP	-0.124 n.s.	0.389 *p* = 0.090	0.576 *p* = 0.008	0.346 n.s.
PTX3	-0.422 *p* = 0.061	0.030 n.s.	0.386 *p* = 0.093	-0.003 n.s.
N vessels	-0.074 n.s.	0.088 n.s.	0.029 n.s.	0.082 n.s.
	CgA_439_/CgA_tot_	CgA-FRs/CgA_tot_	VS-1/CgA_tot_	
ESR	-0.168 n.s.	0.570 *p* = 0.021	0.684 *p* = 0.003	
CRP	-0.036 n.s.	0.254 n.s.	-0.309 n.s.	
PTX3	-0.435 *p* = 0.055	0.159 n.s.	0.480 *p* = 0.032	
N vessels	0.072 n.s.	-0.012 n.s.	-0.057 n.s.	
Hypertensive TA (N = 22)				
	CgA_439_	CgA-FRs	VS-1	CgA_tot_
ESR	0.517 *p* = 0.020	0.295 n.s.	-0.192 n.s.	0.276 n.s.
CRP	0.149 n.s.	0.347 n.s.	0.049 n.s.	0.324 n.s.
PTX3	-0.030 n.s.	0.157 n.s.	0.088 n.s.	0.153 n.s.
N vessels	0.382 *p* = 0.076	-0.222 n.s.	-0.259 n.s.	-0.104 n.s.
	CgA_439_/CgA_tot_	CgA-FRs/CgA_tot_	VS-1/CgA_tot_	
ESR	0.536 *p* = 0.015	-0.085 n.s.	-0.561 *p* = 0.010	
CRP	0.134 n.s.	0.136 n.s.	-0.455 *p* = 0.033	
PTX3	-0.052 n.s.	-0.082 n.s.	-0.010 n.s.	
N vessels	0.389 *p* = 0.073	-0.203 n.s.	-0.311 n.s.	

Levels of CgA_439_, CgA-FRs, VS-1 or their ratios to CgA_tot_ were correlated with markers of systemic inflammation (ESR and CRP), of local inflammation (PTX3) and with the number of vessels involved by the disease (N vessels, see “Methods”). The impact of the treatment with PPIs and of arterial hypertension is also considered. The Spearman correlation coefficient and the relative *p* values are shown

*CgA* chromogranin-A, *TA* Takayasu arteritis, *CgA*_*439*_ full-length CgA (residues 1–439), *CgA-FRs* fragments of CgA spanning from the N-terminus to the central region but lacking the C-terminal region, *VS-1* vasostatin-1, *CgA*_*tot*_ total CgA, *ESR* erythrocyte sedimentation rate, *n.s.* not significant, *CRP* C-reactive protein, *PTX3* pentraxin-3, *PPI* proton-pump inhibitor

In normotensive patients, VS-1 levels correlated positively with the concentration of CRP (Spearman coefficient 0.576, *p* = 0.008), and VS-1/CgA_tot_ correlated with ESR and PTX3 values (Spearman coefficient 0.684, *p* = 0.003 and 0.480, *p* = 0.032, respectively). CgA-FRs positively correlated with ESR (Spearman coefficient 0.570, *p* = 0.021; Table 3 and Additional file 3: Figure S2A-D). In hypertensive patients, CgA_439_ and CgA_439_/CgA_tot_ positively correlated with ESR (Spearman coefficient 0.517, *p* = 0.020 and 0.536, *p* = 0.015, respectively), while VS-1/CgA_tot_ negatively correlated with ESR and CRP (Spearman coefficient -0.561, *p* = 0.010 and -0.455, *p* = 0.033, respectively; Table 3 and Additional file 3: Figure S2E-H). These data suggest that arterial hypertension influences the link between the CgA system and inflammation in TA.

### CgA_439_, CgA-FRs and VS-1 levels do not reflect disease activity, extent or progression

Similar concentrations of CgA_439_, CgA-FRs and VS-1 (Fig. 2 and Table 4) and CgA_439_/CgA_tot_, CgA-FRs/CgA_tot_ and VS-1/CgA_tot_ (data not shown) were observed in patients with active *versus* inactive disease, defined based on NIH criteria [5]. Moreover, levels of CgA_439_, CgA-FRs and VS-1 and their ratios to CgA_tot_ did not correlate with the number of arterial lesions as a measure of disease extent (Table 3). Concentrations of CgA_439_, CgA-FRs and VS-1 were similar in patients regardless of the presence of vascular progression, defined as appearance of novel lesions or of increased thickness and/or length and/or percentage of luminal stenosis of established vasculitic lesions as assessed by imaging follow-up. We assessed the anti-angiogenic CgA potential by summing the ranks within the TA sample of CgA_439_ and VS-1 [17]. We found that the anti-angiogenic CgA potential was unrelated to disease activity, to the number of involved vessels and to vascular progression. The group of patients on PPIs comprises eight out of the nine patients undergoing vascular progression. Vascular progression in patients on PPIs was associated with a significantly reduced anti-angiogenic CgA potential (*p* = 0.01, Table 4). The anti-angiogenic CgA potential was as well significantly reduced in hypertensive TA patients undergoing progression of the vascular damage (*p* = 0.05, Table 4).

**Table 4:** CgA peptides according to the presence of arterial wall enhancement, of active TA and of vascular progression

Whole TA sample	No arterial wall enhancement (N = 25)	Arterial wall enhancement (N = 5)	*p* value
CgA_439_ (nM)	0.06 (0–0.62)	0.00 (0–0.78)	n.s.
CgA-FRs (nM)	1.85 (0.39–5.03)	1.41 (0.32–3.93)	n.s.
VS-1 (nM)	0.25 (0.02–1.15)	0.23 (0.10–0.79)	n.s.
Rank CgA_439_ + rank VS-1	38 (5–83)	36 (24–80)	n.s.
Patients on PPIs	No arterial wall enhancement (N = 20)	Arterial wall enhancement (N = 4)	*p* value
CgA_439_ (nM)	0.07 (0–0.62)	0.00 (0–0.78)	n.s.
CgA-FRs (nM)	2.45 (0.41–5.03)	2.60 (1.25–3.93)	n.s.
VS-1 (nM)	0.27 (0.06-1.15)	0.48 (0.22–0.79)	n.s.
Rank CgA_439_ + rank VS-1	44 (29–83)	32 (24–80)	n.s.
Hypertensive patients	No arterial wall enhancement (N = 13)	Arterial wall enhancement (N = 2)	*p* value
CgA_439_ (nM)	0.06 (0–0.62)	0.43 (0.09–0.78)	n.s.
CgA-FRs (nM)	2.76 (0.77–5.03)	2.06 (0.32–3.8)	n.s.
VS-1 (nM)	0.38 (0.17–1.15)	0.42 (0.10–0.74)	n.s.
Rank CgA_439_ + rank VS-1	50 (28–83)	58 (36–80)	n.s.
Whole TA sample	Inactive TA (N = 30)	Active TA (N = 12)	*p* value
CgA_439_ (nM)	0.05 (0–0.62)	0.03 (0–0.78)	n.s.
CgA-FRs (nM)	1.37 (0.22–6.68)	1.80 (0.28–5.03)	n.s.
VS-1 (nM)	0.19 (0.02–1.15)	0.21 (0.09–0.79)	n.s.
Rank CgA_439_ + rank VS-1	37 (5–83)	35 (18–80)	n.s.
Patients on PPIs	Inactive TA (N = 18)	Active TA (N = 12)	*p* value
CgA_439_ (nM)	0.08 (0–0.62)	0.03 (0–0.78)	n.s.
CgA-FRs (nM)	2.55 (0.41–6.68)	1.80 (0.28–5.03)	n.s.
VS-1 (nM)	0.32 (0.06–1.15)	0.21 (0.09–0.79)	n.s.
Rank CgA_439_ + rank VS-1	52 (24–83)	35 (18–80)	0.072
Hypertensive patients	Inactive TA (N = 15)	Active TA (N = 7)	*p* value
CgA_439_ (nM)	0.08 (0–0.62)	0.06 (0–0.78)	n.s.
CgA-FRs (nM)	2.50 (0.22–6.68)	1.85 (0.28–5.03)	n.s.
VS-1 (nM)	0.39 (0.05–1.15)	0.20 (0.09–0.74)	n.s.
Rank CgA_439_ + rank VS-1	50 (8–83)	39 (18–80)	n.s.
Whole TA sample	No vascular progression (N = 31)	Vascular progression (N = 9)	^p^ value
CgA_439_ (nM)	0.06 (0–0.78)	0 (0–0.14)	n.s.
CgA-FRs (nM)	1.49 (0.22–5.03)	1.76 (0.32–4.24)	n.s.
VS-1 (nM)	0.20 (0.02–1.15)	0.20 (0.09–0.79)	n.s.
Rank CgA_439_+ rank VS-1	42 (5–83)	36 (22–40)	n.s.
Patients on PPIs	No vascular progression (N = 20)	Vascular progression (N = 8)	*p* value
CgA_439_ (nM)	0.09 (0–0.78)	0 (0–0.14)	0.063
CgA-FRs (nM)	2.45 (0.41–5.03)	1.81 (0.46–4.24)	n.s.
VS-1 (nM)	0.27 (0.06–1.15)	0.21 (0.09–0.79)	n.s.
Rank CgA_439_ + rank VS-1	52 (24–83)	34.5 (22–40)	0.010
Hypertensive patients	No vascular progression (N = 16)	Vascular progression (N = 4)	*p* value
CgA_439_ (nM)	0.08 (0–0.78)	0.05 (0–0.14)	n.s.
CgA-FRs (nM)	2.63 (0.22–5.03)	1.40 (0.32–4.24)	n.s.
VS-1 (nM)	0.39 (0.05–1.15)	0.15 (0.09–0.37)	n.s.
Rank CgA_439_ + rank VS-1	52 (8–83)	33 (22–39)	0.050

Levels of CgA_439_, CgA-FRS, VS-1 and the sum of the ranks of CgA_439_ and of VS-1 are compared according to the presence of arterial wall enhancement, of active disease and of vascular progression. The impact of PPI therapy or arterial hypertension is also considered

*CgA* chromogranin-A, *TA* Takayasu arteritis, *CgA*_*439*_ full-length CgA (residues 1–439), *n.s.* not significant, *CgA-FRs* fragments of CgA spanning from the N-terminus to the central region but lacking the C-terminal region, *VS-1* vasostatin-1, *PPI* proton-pump inhibitor

### Immune-modulating agents modulate CgA levels and fragmentation in TA

Patients on prednisone had increased CgA-FRs (Additional file 4: Table S3). The difference was lost after stratification for PPIs (Additional file 4: Table S3) or for arterial hypertension (data not shown). Patients on conventional immunosuppressive agents had higher CgA-FRs, and the difference was maintained after stratification for PPIs (Additional file 4: Table S3) or for arterial hypertension (data not shown). Moreover, patients on immunosuppressive therapy had lower CgA-FRs/CgA_tot_ and a trend towards lower VS-1/CgA_tot_ (Additional file 4: Table S3), suggesting specific regulation of CgA processing. Absolute or relative concentrations of CgA and its peptides were similar in patients with or without TNF blockers or anticoagulant therapies (data not shown).

### Multivariate analysis

To verify the correlation between stratifying variables and plasma levels of CgA peptides, we performed a four-factor analysis of variance (ANOVA) (Additional file 5: Table S2). Plasmatic levels of CgA peptides were related with therapy with PPI, and the VS-1/CgA_tot_*ratio* with treatment with immunosuppressive agents. The relationship between the presence of arterial hypertension and the plasma levels of CgA-FR and VS-1 did not reach the threshold set for statistical significance (*p* = 0.117 and 0.087, respectively), possibly because of the number of factors relatively to the sample size and of the association between arterial hypertension and therapy with immunosuppressive agents (*p* = 0.040, Table 2). Importantly, arterial progression correlated with reduced anti-angiogenic CgA potential (*p* = 0.032) at the four-way ANOVA analysis (Additional file 5: Table S2).

## Discussion

We make some significant observations. First, total CgA and many bioactive CgA-derived peptides are increased in TA patients. Second, inter-patient variability indicates that the CgA system is modulated in TA. Correlation studies show that variables related to disease phenotype (e.g. arterial hypertension), anti-rheumatic drugs and other supportive medications (e.g. PPIs) influence the CgA system and impact on its relationship with inflammation. CgA-derived peptides have been proved to be biologically active in vivo and to influence vascular events involved in the pathogenesis of TA and we observed a reduced anti-angiogenic CgA potential in patients undergoing vascular progression. The resulting effect of pro- and anti-angiogenic CgA forms is difficult to predict in TA patients, and further studies are needed to confirm these preliminary observations. However, our findings raise the possibility that therapies commonly used in patients with vasculitis may indirectly influence vascular events by modulating the CgA system.

Vasa vasorum neongiogenesis is typical of large vessel vasculitides [11, 12]. Inflammatory cells infiltrate vasculitic lesions through vasa vasorum, and neoangiogenesis within arterial lesions have been associated with histologic features, such as disruption of the internal elastic membrane and intimal hyperplasia, which is thought to cause stenosis [11, 30]. The CgA system modulates neoangiogenesis [15, 17] and fibroblast adhesion, endothelial and VSMC proliferation and migration, and endothelial response to inflammatory stimuli.

PPIs raise CgA levels by inducing gastric entherochromaffin cell hyperplasia as a consequence of hypochlorhydia. We found that patients treated with PPIs had increased levels of CgA-FRs and VS-1, even if the CgA processing is apparently conserved. PPI treatment is common in TA because of the use of steroids, anticoagulants and/or anti-platelet agents. Our data raise the possibility that PPIs indirectly influence vascular events in TA *via* the CgA system. It is difficult to predict whether the final effect of PPIs would be vaso-protective or not, because different CgA-derived peptides have opposite actions with non-linear concentration-response curves [15, 17]. Over-prescription of acid secretion-blocking therapies is frequent, and we suggest limiting these agents to TA patients with a clear indication or using other gastro-protective agents until further prospective studies on larger cohorts clarify effects of PPIs on vascular biology. In this regard, TA would represent a paradigmatic condition, as poorly known effects of common medications are likely to occur also in other diseases. Therapy with non-biologic immunosuppressive agents was associated with regulation of the CgA system and an altered processing of CgA. It would be important to understand the mechanisms of this effect and whether this may be helpful or detrimental in large vessel vasculitis.

We found that arterial hypertension was associated with higher levels of CgA-FR and VS-1 in TA, and that a normotensive or hypertensive status influenced the link between the CgA system and inflammation in TA. Arterial hypertension has a complex relationship with the CgA system, as hypertensive patients have higher levels of total CgA [31], but deletion of the CgA gene causes development of arterial hypertension in mice [32]. Higher adrenergic tone and CgA release in hypertensive patients, together with a negative feedback of CgA-derived peptides (such as catestatin on sympathetic terminals), are believed to explain such findings [33, 34]. In TA, concurrent inflammation and vascular injury adds another level of complexity. Arterial hypertension is frequent in TA and identifies patients with a worse outcome [7]. Specific disease-related mechanisms are supposed to participate in arterial hypertension in these patients [6]: renal hypoperfusion due to renal artery stenosis or atypical aortic coarctation, reduction of total arterial compliance due to widespread arterial involvement, baroreceptor dysfunction due to aortic arch or supra-aortic involvement, and steroid side effects. In this context, our data suggest a potential multi-directional interference between arterial hypertension, inflammation and vascular involvement.

This study has limitations: first, we used imaging and pentraxin-3 rather than histology for assessing vascular inflammation, since surgery is rarely performed in TA. Second, TA is a rare disease and large cohorts are difficult to establish. This is an observational study and the relatively small number of patients and the confounding effects of disease- and treatment-associated variables might limit its statistical power. Despite these limitations, a statistically significant inverse association has been detected between the levels of anti-angiogenic CgA peptides and vascular progression in the more active TA patients on PPI. A similar inverse association has been detected in TA patients with arterial hypertension. These results might reflect a protective action of signals limiting angiogenesis associated to the arterial wall, which could as well restrict the maladaptive vascular remodelling that is the hallmark of TA. Further prospective studies on larger groups of patients are warranted to verify whether this is indeed the case.

## Conclusions

We observed marked alterations in TA of the circulating levels of CgA-derived polypeptides that might have an effect on the vascular biology. Both disease- and therapy-related variables influence the blood levels of CgA-derived peptides. Until further study clarifies the potential role of CgA-derived polypeptides in large vessel vasculitis, care should be taken with correct prescription of anti-rheumatic and supportive medications, such as PPIs.

## Abbreviations

CgA, chromogranin-A; CgA_439_, full-length CgA (residues 1–439); CgA-FRs, fragments of CgA spanning from the N-terminus to the central region but lacking the C-terminal region, CgA_tot_, total CgA; CRP, C-reactive protein; CTA, computed tomography angiography; ESR, erythrocyte sedimentation rate; GCA, giant cell arteritis; IL, interleukin; HCs, healthy controls; MRA, magnetic resonance angiography; PPI, proton-pump inhibitors; PTX3, pentraxin-3; TA, Takayasu arteritis; TNF, tumour necrosis factor; US, ultrasonography; VS-1, vasostatin-1; VSMCs, vascular smooth muscle cells

## Additional files

Additional file 1: Figure S1. Schematic representation of the CgA peptides and ELISA tests used in the study. (JPG 230 kb) Additional file 2: Table S1. CgA peptides in TA patients with and without arterial hypertension, stratified for therapy with PPIs. (DOC 49 kb) Additional file 3: Figure S2. Arterial hypertension influences the link between the CgA system and inflammation in TA. Correlations between CgA_439_, CgA-FRs, and VS-1 or their ratios to CgA_tot_, and acute-phase markers ESR, CRP and PTX3, in normotensive (*panels A-D*) or in hypertensive patients (*panels E-H*). Significant correlations only are shown. (JPG 563 kb) Additional file 4: Table S3. CgA peptides in patients with TA stratified for therapy with steroids or immunosuppressive agents. (DOC 63 kb) Additional file 5: Table S2. Four-way analysis of the variance (ANOVA) of the levels of CgA peptides and of the anti-angiogenic CgA potential. (DOC 44 kb)
