# Supplementary figures and images for: Chromogranin-A production and fragmentation in patients with Takayasu arteritis
Source: Arthritis Res Ther. 2016 Aug 17;18:187. doi: 10.1186/s13075-016-1082-2 (PMC4987982; doi:10.1186/s13075-016-1082-2)

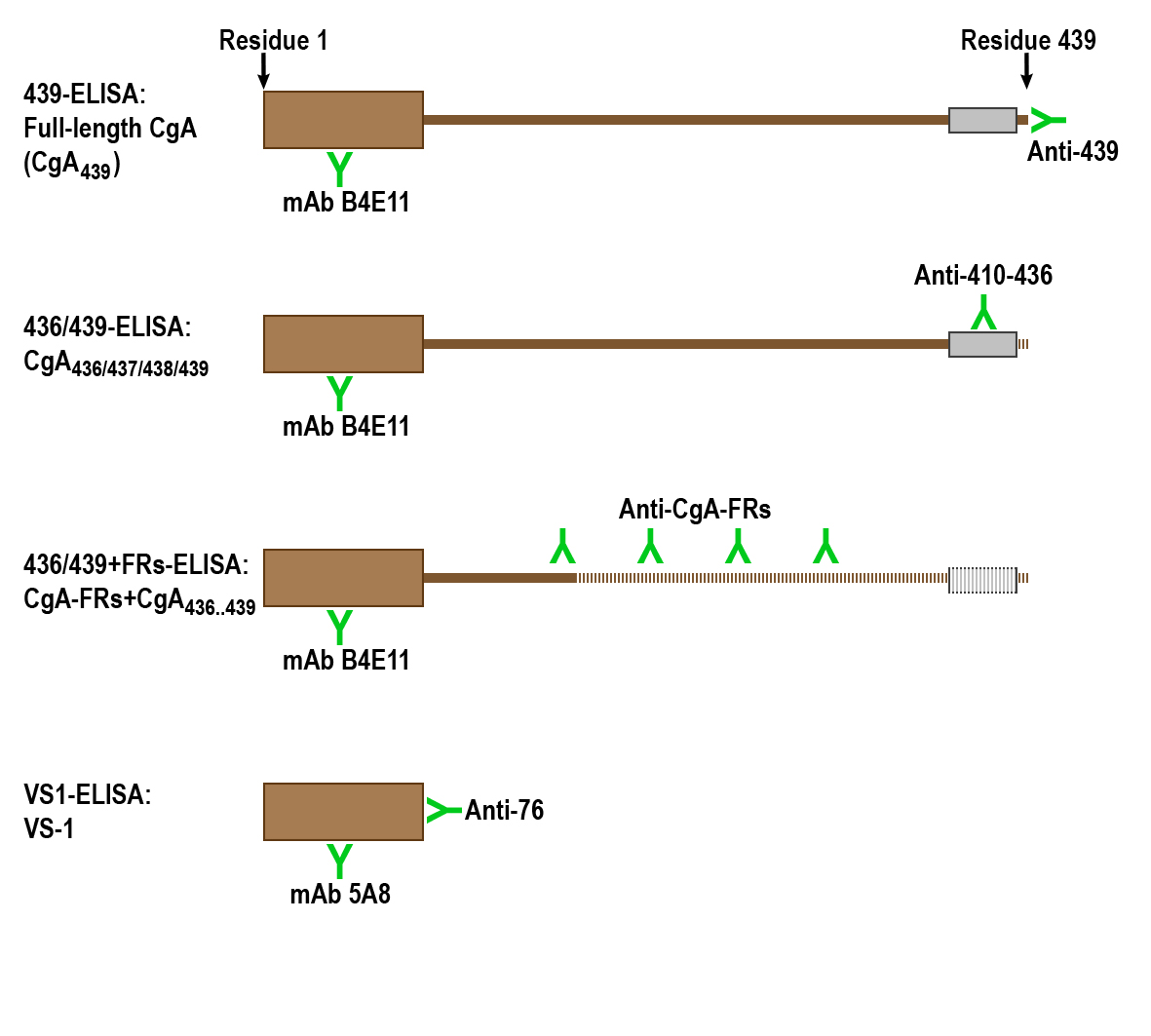

Supplement: Additional file 1: Figure S1. — Schematic representation of the CgA peptides and ELISA tests used in the study. (JPG 230 kb) [file 13075_2016_1082_MOESM1_ESM.jpg]

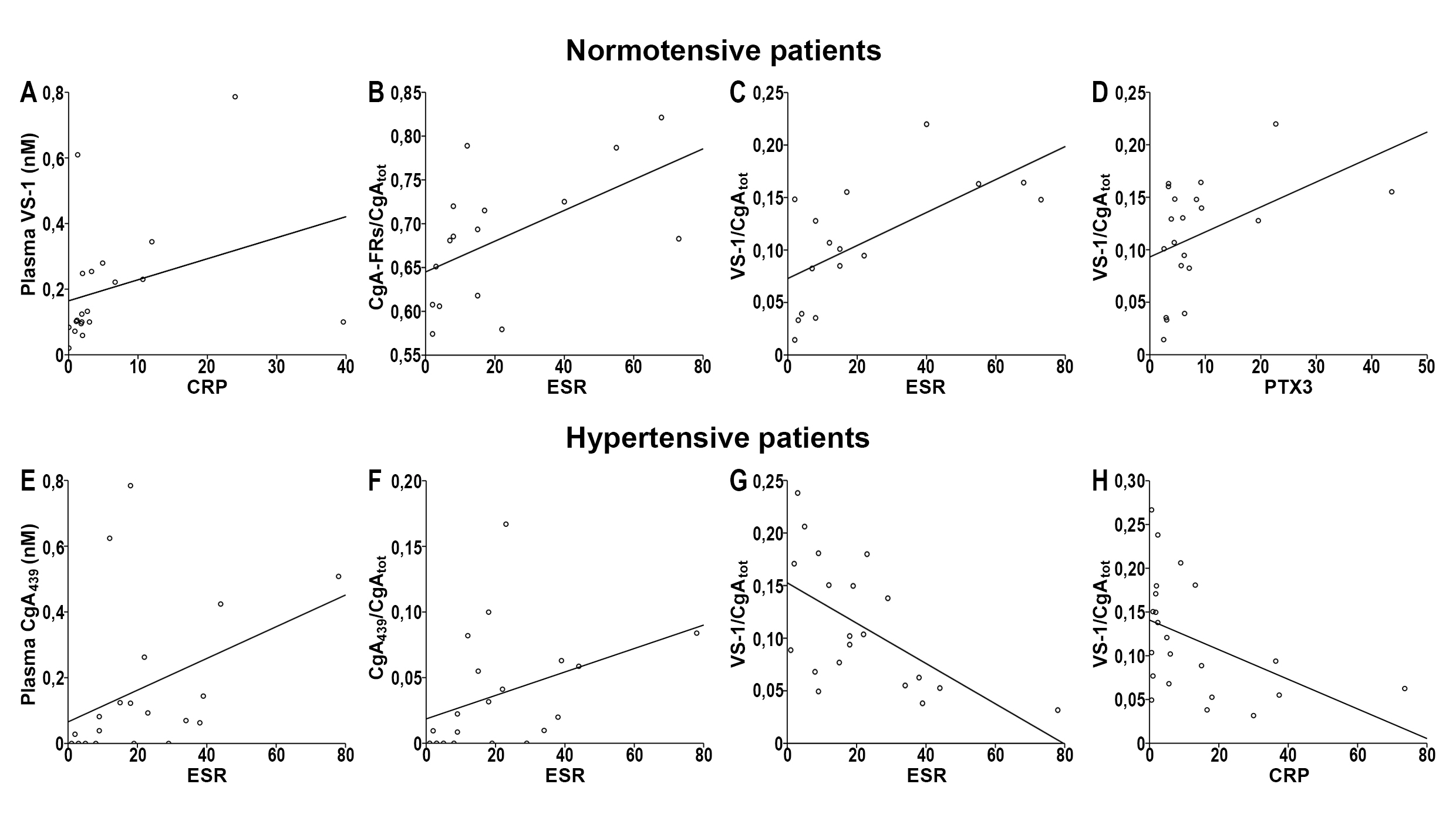

Supplement: Additional file 3: Figure S2. — Arterial hypertension influences the link between the CgA system and inflammation in TA. Correlations between CgA439, CgA-FRs, and VS-1 or their ratios to CgAtot, and acute-phase markers ESR, CRP and PTX3, in normotensive (panels A-D) or in hypertensive patients (panels E-H). Significant correlations only are shown. (JPG 563 kb) [file 13075_2016_1082_MOESM3_ESM.jpg]
