# Supplementary material for: Chromogranin-A production and fragmentation in patients with Takayasu arteritis
Source: Arthritis Res Ther. 2016 Aug 17;18:187. doi: 10.1186/s13075-016-1082-2 (PMC4987982; doi:10.1186/s13075-016-1082-2)
Supplement: Additional file 2: Table S1. — CgA peptides in TA patients with and without arterial hypertension, stratified for therapy with PPIs. (DOC 49 kb) [file 13075_2016_1082_MOESM2_ESM.doc]

**Table S1. CgA peptides in TA patients with and without arterial hypertension, stratified for therapy with PPIs**

|  | **Arterial hypertension** | | |  | |
| --- | --- | --- | --- | --- | --- |
| **Patients without PPIs (N=12)** | **No (N=8)** | **Yes (N=4)** | | **p-value** | |
| Total CgA (nM) | 1.12 (0.54-2.66) | | 0.79 (0.45-1.1) | | n.s. |
| CgA_439_ (nM) | 0 (0-.19) | | 0.04 (0-0.09) | | n.s. |
| CgA-FRs (nM) | 0.79 (0.39-1.82) | | 0.54 (0.22-0.86) | | n.s. |
| VS-1 (nM) | 0.10 (0.02-0.34) | | 0.09 (0.05-0.26) | | n.s. |
| CgA_439_/CgA_tot_ | 0% (0-15%) | | 8% (0-18%) | | n.s. |
| CgA-FRs/CgA_tot_ | 70% (61-80%) | | 64% (50-84%) | | n.s. |
| VS-1/CgA_tot_ | 12% (1-16%) | | 15% (9-24%) | | n.s. |
| Rank CgA_439_+ rank VS-1 | 22 (5-50) | | 27.5 (8-36) | | n.s. |
|  | **Arterial hypertension** | | | |  |
| **Patients on PPIs (N=12)** | **No (N=12)** | | **Yes (N=18)** | | **p-value** |
| Total CgA (nM) | 2.08 (0.68-6.31) | | 4.43 (0.60-7.85) | | 0.017* |
| CgA_439_ (nM) | 0.03 (0-0.44) | | 0.08 (0-0.78) | | n.s. |
| CgA-FRs (nM) | 1.33 (0.41-3.93) | | 2.82 (0.28-6.68) | | 0.035* |
| VS-1 (nM) | 0.23 (0.06-0.79) | | 0.39 (0.09-1.15) | | 0.087 |
| CgA_439_/CgA_tot_ | 0% (0-24%) | | 2% (0-18%) | | n.s. |
| CgA-FRs/CgA_tot_ | 68% (57-82%) | | 64% (47-94%) | | n.s. |
| VS-1/CgA_tot_ | 13% (1-22%) | | 11% (1-27%) | | n.s. |
| Rank CgA_439_+ rank VS-1 | 34.5 (24-64) | | 52 (18-83) | | 0.022* |
