# Supplementary material for: Chromogranin-A production and fragmentation in patients with Takayasu arteritis
Source: Arthritis Res Ther. 2016 Aug 17;18:187. doi: 10.1186/s13075-016-1082-2 (PMC4987982; doi:10.1186/s13075-016-1082-2)
Supplement: Additional file 4: Table S3. — CgA peptides in patients with TA stratified for therapy with steroids or immunosuppressive agents. (DOC 63 kb) [file 13075_2016_1082_MOESM4_ESM.doc]

**Table S3. CgA peptides in patients with TA stratified for therapy with steroids or immunosuppressive agents.**

| **Panel A - all TA patients** | **Without PDN (N=12)** | **On PDN (N=30)** | **p-value** |
| --- | --- | --- | --- |
| CgA_tot_ (nM) | 1.06 (0.45-7.61) | 2.91 (0.60-7.85) | 0.007** |
| CgA_439_ (nM) | 0.09 (0-0.62) | 0.03 (0-0.78) | n.s. |
| CgA-FRs (nM) | 0.76 (0.22-3.93) | 1.63 (0.22-6.68) | 0.009** |
| VS-1 (nM) | 0.11 (0.02-1.15) | 0.20 (0.02-1.15) | 0.060 |
| CgA_439_/CgA_tot_ | 7% (0-18%) | 1% (0-24%) | n.s. |
| CgA-FRs/CgA_tot_ | 68% (48-82%) | 66% (47-94%) | n.s. |
| VS-1/CgA_tot_ | 14% (1-24%) | 11% (1-27%) | n.s. |
| **Panel B - patients on PPIs** | **Without PDN (N=3)** | **On PDN (N=27)** | **p-value** |
| CgA_tot_ (nM) | 4.79 (2.26-7.61) | 3.16 (0.60-7.85) | n.s. |
| CgA_439_ (nM) | 0.12 (0-0.62) | 0.06 (0-0.78) | n.s. |
| CgA-FRs (nM) | 3.69 (1.49-3.93) | 1.94 (0.28-6.68) | n.s. |
| VS-1 (nM) | 0.78 (0.17-1.15) | 0.25 (0.06-1.00) | n.s. |
| CgA_439_/CgA_tot_ | 5% (0-8%) | 2% (0-24%) | n.s. |
| CgA-FRs/CgA_tot_ | 66% (48-82%) | 65% (47-94%) | n.s. |
| VS-1/CgA_tot_ | 15% (8-16%) | 10% (3-27%) | n.s. |
| **Panel C - all TA patients** | **Without IS agents (N=12)** | **On IS agents (N=30)** | **p-value** |
| CgA_tot_ (nM) | 1.07 (0.45-4.79) | 2.91 (0.54-7.85) | 0.017* |
| CgA_439_ (nM) | 0 (0-0.17) | 0.07 (0-0.78) | n.s. |
| CgA-FRs (nM) | 0.83 (0.22-3.93) | 1.86 (0.28-6.68) | 0.032* |
| VS-1 (nM) | 0.18 (0.05-0.79) | 0.20 (0.02-1.15) | n.s. |
| CgA_439_/CgA_tot_ | 0% (0-24%) | 2% (0-17%) | n.s. |
| CgA-FRs/CgA_tot_ | 72% (50-84%) | 65% (47-94%) | 0.040* |
| VS-1/CgA_tot_ | 13% (8-24%) | 10% (1-27%) | 0.060 |
| **Panel D - Patients on PPIs** | **Without IS agents (N=7)** | **On IS agents (N=23)** | **p-value** |
| CgA_tot_ (nM) | 1.56 (0.69-4.79) | 3.88 (0.54-7.85) | 0.042* |
| CgA_439_ (nM) | 0 (0-0.17) | 0.06 (0-0.78) | n.s. |
| CgA-FRs (nM) | 1.23 (0.41-3.93) | 2.50 (0.28-6.68) | 0.061 |
| VS-1 (nM) | 0.25 (0.06-0.79) | 0.25 (0.09-1.15) | n.s. |
| CgA_439_/CgA_tot_ | 0% (0-24%) | 2% (0-10%) | n.s. |
| CgA-FRs/CgA_tot_ | 72% (57-82%) | 64% (47-94%) | n.s. |
| VS-1/CgA_tot_ | 15% (8-22%) | 10% (3-27%) | 0.061 |
| Concentrations of CgA_439_, CgA-FRS and VS-1 and their ratios to CgA_tot_ in all patients with TA (Panels A and C) and in those on PPIs (Panels B and D) stratified for therapy with steroids and immunosuppressive agents. PDN: prednisone. | | | |
