# Supplementary material for: Chromogranin-A production and fragmentation in patients with Takayasu arteritis
Source: Arthritis Res Ther. 2016 Aug 17;18:187. doi: 10.1186/s13075-016-1082-2 (PMC4987982; doi:10.1186/s13075-016-1082-2)
Supplement: Additional file 5: Table S2. — Four-way analysis of the variance (ANOVA) of the levels of CgA peptides and of the anti-angiogenic CgA potential. (DOC 44 kb) [file 13075_2016_1082_MOESM5_ESM.doc]

**Table S2. Four-way analysis of the variance (ANOVA) of the levels of CgA peptides and of the anti-angiogenic CgA potential.**

| **Dependant variables** | **PPI therapy**  **(p-value)** | **AH**  **(p-value)** | **Vascular progression**  **(p-value)** | **IS**  **(p-value)** |
| --- | --- | --- | --- | --- |
| CgA_439_ | 0.147 | 0.433 | 0.084 | 0.195 |
| CgA-FRs | **0.001** | 0.117 | 0.552 | 0.149 |
| VS-1 | **0.015** | 0.087 | 0.479 | 0.995 |
| CgA_439_/CgA_tot_ | 0.400 | 0.757 | 0.990 | 0.569 |
| CgA-FRs/CgA_tot_ | 0.421 | 0.496 | 0.157 | 0.067 |
| VS-1/CgA_tot_ | 0.488 | 0.322 | 0.732 | **0.022** |
| Rank CgA_439_+ rank VS-1 | **<0.001** | 0.100 | **0.032** | 0.231 |
| AH: arterial hypertension; IS: therapy with immunosuppressive agents. | | | | |
